# Heterogeneity of COVID-19 symptoms and associated factors: Longitudinal analysis of laboratory-confirmed COVID-19 cases in San Antonio

**DOI:** 10.1371/journal.pone.0295418

**Published:** 2023-12-08

**Authors:** Byeong Yeob Choi, Abigail R. Grace, Jack Tsai

**Affiliations:** 1 Joe R. & Teresa Lozano Long School of Medicine, University of Texas Health San Antonio, San Antonio, Texas, United States of America; 2 School of Public Health, University of Texas Health Science Center at Houston, Houston, Texas, United States of America; King Faisal University, SAUDI ARABIA

## Abstract

Few studies have examined heterogeneous associations of risk factors with Coronavirus Disease-2019 (COVID-19) symptoms by type. The objectives of this study were to estimate the prevalence of and risk factors associated with COVID-19 symptoms and to investigate whether the associations differ by the type of symptoms. This study obtained longitudinal data over 6 months from laboratory-confirmed COVID-19 cases in a citywide sample in San Antonio. Sixteen symptoms of COVID-19 infection, measured at baseline and three follow-up times (1, 3, and 6 months), were analyzed using generalized estimating equations (GEE) to investigate potential risk factors while accounting for the repeated measurements. The risk factors included time in months, sociodemographic characteristics, and past or current medical and psychiatric conditions. To obtain interpretable results, we categorized these sixteen symptoms into five categories (cardiopulmonary, neuro-psychological, naso-oropharyngeal, musculoskeletal, and miscellaneous). We fitted GEE models with a logit link using each category as the outcome variable. Our study demonstrated that the associations were heterogeneous by the categories of symptoms. The time effects were the strongest for naso-oropharyngeal symptoms but the weakest for neuro-psychological symptoms. Female gender was associated with increased odds of most of the symptoms. Hispanic ethnicity was also associated with higher odds of neuro-psychological, musculoskeletal, and miscellaneous symptoms. Depression was the most robust psychiatric condition contributing to most of the symptoms. Different medical conditions seemed to contribute to different symptom expressions of COVID-19 infection.

## Introduction

Severe acute respiratory syndrome coronavirus 2 (SARS-CoV-2) or Coronavirus Disease-2019 (COVID-19) has led to a pandemic that has infected hundreds of millions of individuals and has been attributed to millions of deaths around the world [1]. Individuals who have been infected have reported a range of different symptoms, with some symptoms lasting weeks or months after recovery [2]. Symptoms have been heterogeneous in their time frame and presentation. Infected individuals have reported all body symptoms with some of the most common complaints including mental and cognitive problems, chest pain, headache, cough, altered taste and smell, diarrhea, and other gastrointestinal symptoms [3–5].

There is a growing body of literature focused on persistent, residual symptoms of COVID-19 infection termed “Long COVID” [5], and the National Institutes of Health have funded a number of large projects to examine Long COVID and their outcomes. However, there has been inadequate examination of not only Long COVID, but overall, what and which symptoms of COVID-19 are experienced, how they cluster together, and how they vary in timeframes. Moreover, it is not clear how these different dimensions of COVID-19 symptoms are associated with other medical and psychosocial factors.

Studies have documented a range of clinical and psychosocial correlates of COVID-19 symptoms, particularly correlations with symptom severity. For example, reviews have reported that disease severity is greater in older adults, individuals with multiple pre-existing medical comorbidities, or individuals with certain lab result indicators such as high C-reactive protein and lactate dehydrogenase [6, 7]. However, there has been a lack of specificity in tracking different symptom expressions of COVID-19, specifically whether they vary by sociodemographic and clinical characteristics of infected individuals [8].

The objectives of this study were to estimate the prevalence of and risk factors associated with COVID-19 symptoms and to investigate whether these associations differ by the type of COVID-19 symptoms. Post-acute COVID-19 symptoms are heterogeneous, management differs by organ-specific sequelae, and prioritization may be considered for those at high risk for long COVID [9]. Factors such as female sex, minor ethnicity, and comorbidity may contribute to development of long COVID [2, 9–16]. However, the contributions of the risk factors might differ in a symptom-specific way, which our study investigates, and to our knowledge no studies have extensively assessed how these associations may vary. Understanding the heterogeneity of COVID-19 symptoms will improve the identification and management of high-risk groups of COVID-19 survivors who can develop long-COVID. To this end, the current study uses a city-wide sample in a large U.S. city of adults with lab-confirmed cases of COVID-19 and followed them over 6 months to examine patterns of COVID-19 symptom expression. The study builds on previous cross-sectional studies [17] to enhance our understanding of the heterogeneity of COVID-19 symptoms.

## Methods

**Fig 1:**
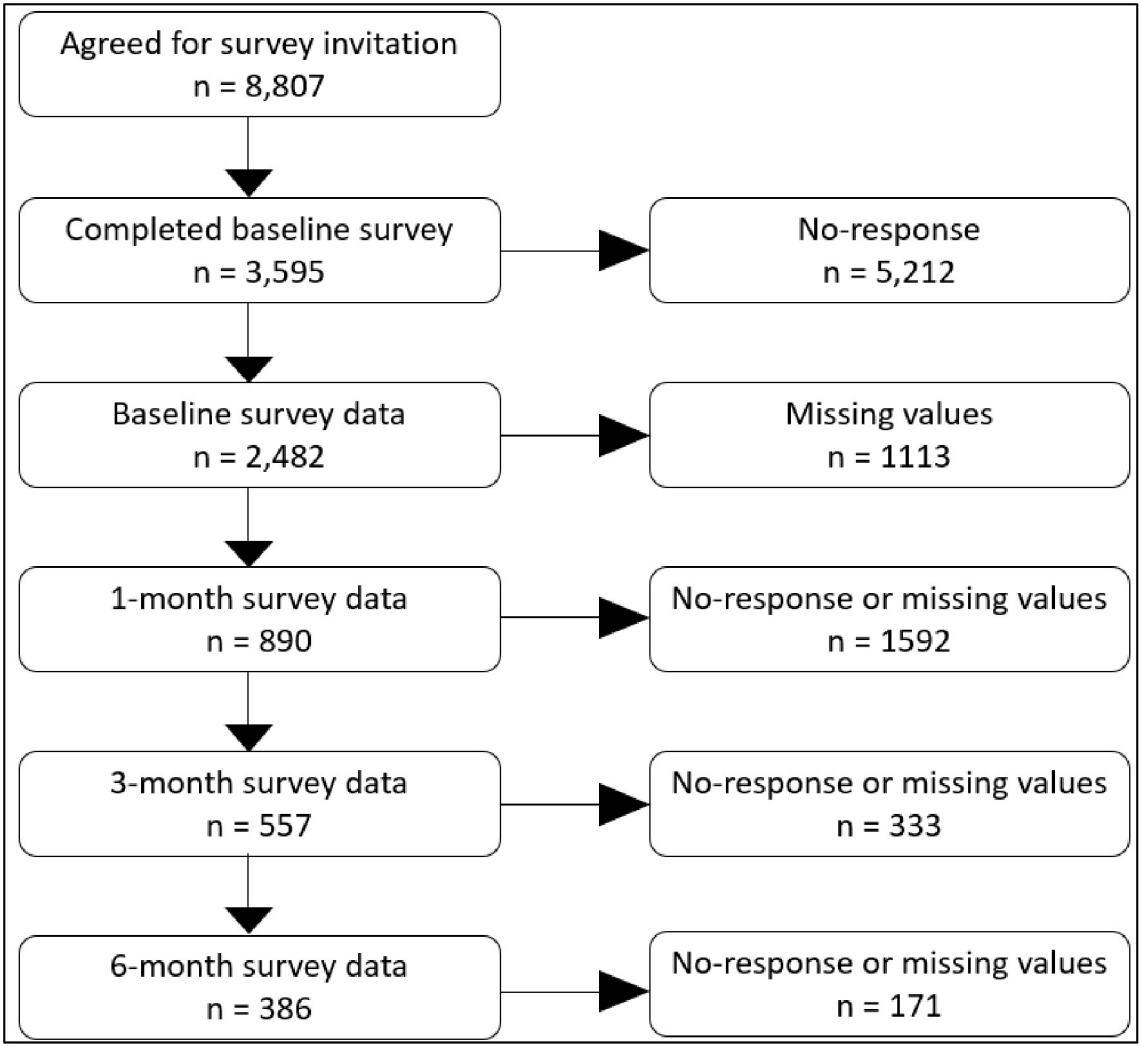
Flow diagram of participants at baseline, 1 month, 3 months, and 6 months.

From 2020–2022, the San Antonio campus for the University of Texas Health Science Center at Houston (UTHealth) School of Public Health led a citywide COVID-19 contact tracing operation in partnership with the City of San Antonio Metropolitan Health District. During that time, the UTHealth School of Public Health contact tracing team initiated over 80,000 calls in San Antonio to contact individuals infected with laboratory-confirmed cases of COVID-19 sent by hospitals and healthcare providers. The contact tracing team gathered personal information from infected individuals about places they visited and people they may have exposed while they were infected. From February 18, 2021 to March 28, 2022, the UTHealth School of Public Health contact tracing team recruited individuals infected with COVID-19 to participate in a longitudinal research study to assess their health and well-being [18, 19]. Follow-up surveys were hosted by the university’s Qualtrics account, and all participants provided informed consent to enroll in the study after completing their contact tracing interview. Participants were provided $10 compensation per assessment. Eligibility criteria for participation was that participants had to be 18 years or older; currently living in San Antonio; have a laboratory-confirmed case of COVID-19 as verified by the contact tracing team; and could read and write in English. All participants provided online written informed consent and study procedures were approved by the institutional review board at UTHealth School of Public Health (IRB# HSC-SPH-20-0931). A total of 8,807 individuals agreed to be sent a survey invitation, and 3,595 (40.8%) participants completed the baseline survey. A flow diagram in Fig 1 shows the numbers of participants used for analysis at four measurement occasions (baseline, 1-month, 3-month, and 6-month).

### Measures

Baseline demographic characteristics were collected, including gender, race/ethnicity, age, education, marital status, and annual income.

**Fig 2:**
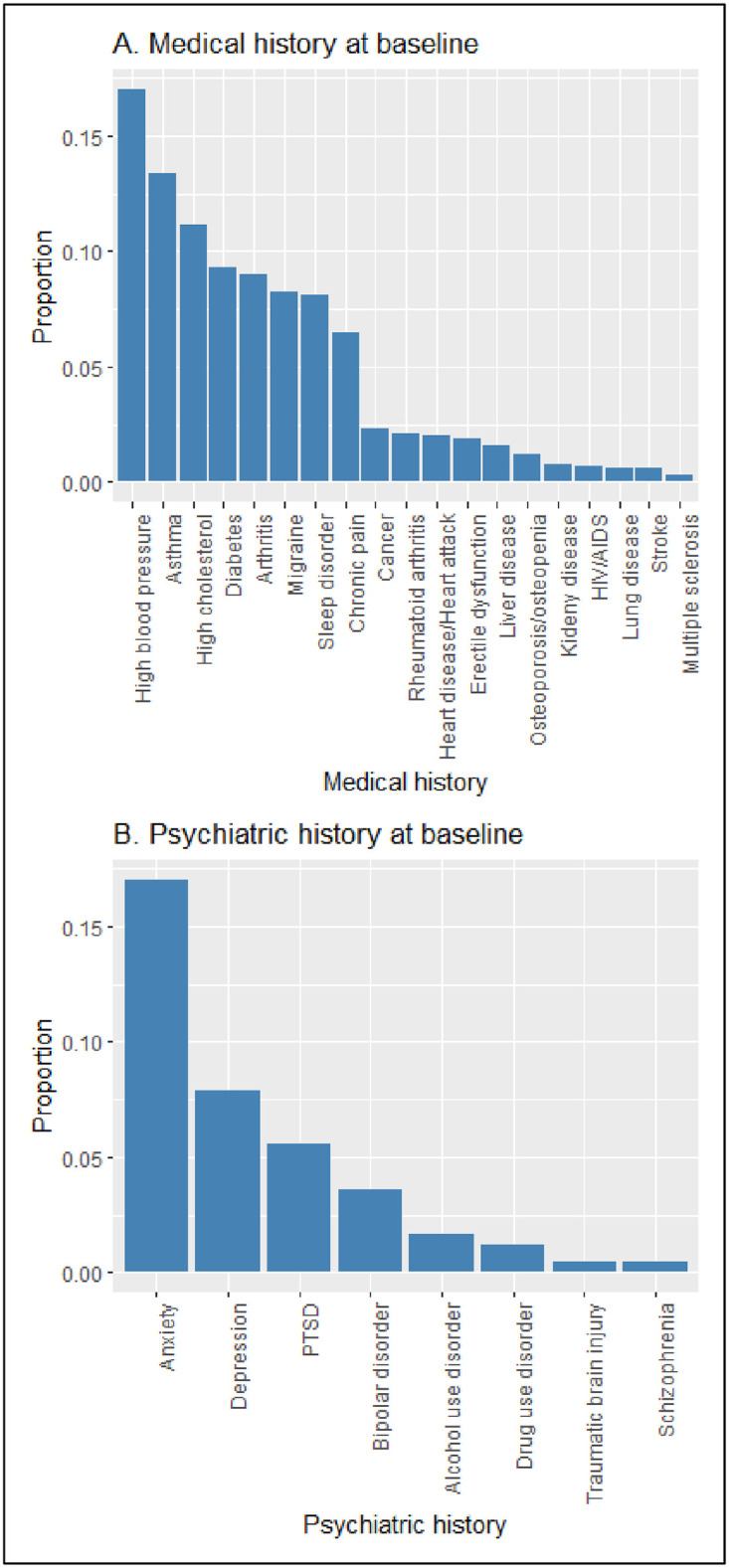
Medical and psychiatric history at baseline.

Medical conditions were assessed by self-report and included arthritis, asthma, cancer, chronic pain, diabetes, erectile dysfunction, heart disease, HIV/AIDS, lung disease, liver disease, high cholesterol, high blood pressure, kidney disease, migraine, multiple sclerosis, osteoporosis, obesity, rheumatoid arthritis, sleep disorder, and stroke (Fig 2).

Psychiatric conditions were also assessed by self-report and included schizophrenia, post-traumatic stress disorder (PTSD), alcohol use, bipolar disorder, anxiety, depression, drug use, and traumatic brain injury (Fig 2).

Sixteen symptoms after testing positive for COVID-19 were measured, but these symptoms were classified into the following five categories, following Aiyegbusi et al. [4].

- Cardiopulmonary: Fatigue, Shortness of breath, Heart palpitations, and Chest pain
- Neuro-psychological: Brain fog, Sleep issues, Depression, and Mood changes
- Naso-oropharyngeal: Cough, and Lack of smell and taste
- Musculoskeletal: Joint pain and Muscle pain
- Miscellaneous: Headache, Hair loss, Fever, and Rash

### Data analysis

Descriptive statistics were used to summarize the baseline characteristics of participants. Categorical variables were summarized with counts and percentages. Continuous variables were summarized with means and standard deviations. Medians and ranges were also used to summarize continuous variables.

We plotted the percentages of participants who experienced each category of the symptoms over four measurement occasions (baseline, 1 month, 3 months, and 6 months) to observe the population burden of each category over time.

To examine the associations, we conducted a series of generalized estimating equation (GEE) analyses [20] based on the longitudinal data of laboratory-confirmed COVID-19 cases in San Antonio. Instead of modeling individual symptoms, we used the five categories as model outcomes to obtain concise and interpretable results. Accordingly, each model outcome indicated whether participants experienced at least one of the individual symptoms within the category. The GEE models included demographic characteristics and medical and psychiatric history. The models also had time in months and the square of time to account for potential nonlinear time effects. We used a logit link to estimate the odd ratios for the predictors and an unstructured covariance matrix to account for repeated measurements.

Among the predictors, some medical and psychiatric conditions in the past were rare in our study population. Including these rare events as predictors can cause model fits to be unstable due to the separation problem [21], and these events might not be relevant to the overall population. Therefore, we selected medical and psychiatric conditions if their proportions at baseline were at least 5%. As a result, we selected arthritis, asthma, chronic pain, diabetes, high cholesterol, high blood pressure, migraine, and sleep disorder among the 21 medical conditions. Out of the 10 psychiatric conditions, we selected anxiety, depression, and PTSD.

We used R version 4.2.2 [22] to generate study results and the R package ‘gee’ [23] to fit the GEE models. The threshold for statistical significance was a 2-sided p-value of 0.05.

## Results

### Baseline results

Our analysis focused on the participants with complete information on all considered variables and baseline data. As a result, the analysis data comprised 2482, 890, 557, and 386 observations at baseline, 1, 3, and 6 months (Fig 1). Thus, the total of 4315 observations were used for statistical analysis. Table 1 shows the demographics of participants at baseline. Most participants were female, White Hispanic or non-Hispanic, employed, single or married/living with a partner, and had at least some college education and an annual income below $60,000.

**Table 1:** Baseline demographic characteristics among adults with COVID-19 infection.

	(N = 2482)
Gender	
Female	1657 (66.8%)
Male	825 (33.2%)
Race	
White Non-Hispanic	660 (26.6%)
White Hispanic	1399 (56.4%)
Black Non-Hispanic	215 (8.7%)
Black Hispanic	58 (2.3%)
Asian/Pacific Islander	67 (2.7%)
Other	83 (3.3%)
Age	
Mean (SD)	36.1 (12.5)
Median [Min, Max]	33.0 [18.0, 84.0]
Education	
Below high school	64 (2.6%)
High school/GED	578 (23.3%)
Some college	762 (30.7%)
Associates/Bachelors	804 (32.4%)
Master’s degree	223 (9.0%)
Doctoral degree	51 (2.1%)
Marital status	
Single	1106 (44.6%)
Married/Living with partner	1102 (44.4%)
Divorced/Separated/Widowed	274 (11.0%)
Employment	
Employed full/half-time	1811 (73.0%)
Unemployed/Other	400 (16.1%)
Disabled/Retired	138 (5.6%)
Self-employed	133 (5.4%)
Income	
No income	235 (9.5%)
$1-$19,999	535 (21.6%)
$20,000-$39,999	670 (27.0%)
$40,000-$59,999	492 (19.8%)
$60,000-$79,999	260 (10.5%)
$80,000-$99,999	128 (5.2%)
$100,000+	162 (6.5%)

In Fig 2, we displayed the proportions of participants who had any past medical and psychiatric conditions at baseline. Before SARS-CoV-2 infection, seventeen percent of participants had high blood pressure, followed by asthma/chronic bronchitis/COPD (13.4%), high cholesterol (11.1%), diabetes (9.3%), arthritis (9.0%), migraine (8.3%), sleep disorder (8.1%), and chronic pain (6.4%). As for psychiatric history, 17.0% of participants experienced anxiety, followed by depression (7.9%) and PTSD (5.6%).

**Fig 3:**
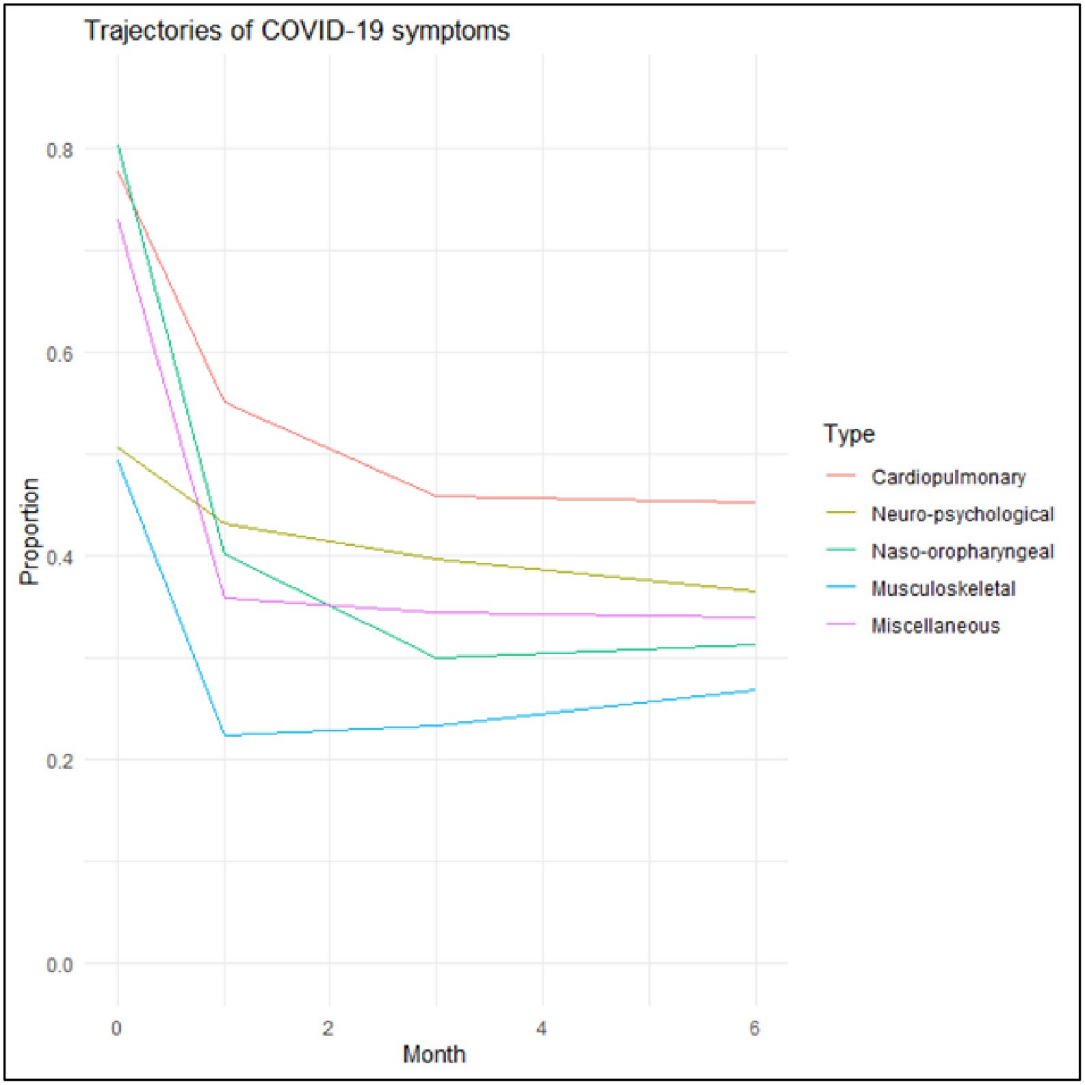
Trajectories of the five categories of COVID-19 symptoms over 6 months.

Fig 3 shows how the proportion of participants who experienced each category of symptoms changed nonlinearly over time. Over 70% of participants experienced cardiopulmonary, naso-oropharyngeal, and miscellaneous symptoms at baseline. Approximately half of the participants experienced neuro-psychological and musculoskeletal symptoms at baseline, but neuro-psychological symptoms persisted over time. Among the five categories, cardiopulmonary symptoms were the most prevalent over time.

### Regression analysis results

The GEE results were presented in Table 2, which shows the ORs for the baseline demographic characteristics, medical, and psychiatric history. The predictors listed below had significant associations with the symptoms in the GEE models at a significance level of 5%. The linear and quadratic terms for time were significant for all categories, and therefore not listed below.

- Cardiopulmonary: gender, education, income, arthritis, asthma, high blood pressure, migraine, anxiety, depression, and PTSD.
- Neuro-psychological: gender, race/ethnicity, age, income, asthma, chronic pain, migraine, sleep disorder, anxiety, depression, and PTSD.
- Naso-oropharyngeal: gender, race/ethnicity, education, income, arthritis, and diabetes.
- Musculoskeletal: race/ethnicity, marital status, income, arthritis, chronic pain, and depression.
- Miscellaneous: gender, race/ethnicity, age, employment, arthritis, chronic pain, migraine, and depression.

**Table 2:** Multivariable odds ratios relating time, baseline demographic, and medical and psychiatric history to COVID-symptoms for 6 months.

	Cardiopulmonary		Neuro-psychological		Naso-oropharyngeal		Musculoskeletal		Miscellaneous	
	OR	p-value	OR	p-value	OR	p-value	OR	p-value	OR	p-value
Time	**0.415**	**0.000**	**0.787**	**0.000**	**0.229**	**0.000**	**0.401**	**0.000**	**0.317**	**0.000**
Time^2^	**1.112**	**0.000**	**1.025**	**0.007**	**1.208**	**0.000**	**1.136**	**0.000**	**1.163**	**0.000**
Gender										
Female	1.000	NA	1.000	NA	1.000	NA	1.000	NA	1.000	NA
Male	**1.456**	**0.000**	**1.645**	**0.000**	**1.300**	**0.002**	1.025	0.770	**1.280**	**0.003**
Race/ethnicity										
White Non-Hispanic	1.000	NA	1.000	NA	1.000	NA	1.000	NA	1.000	NA
White Hispanic	1.050	0.625	**1.357**	**0.001**	1.084	0.398	1.186	0.062	**1.216**	**0.035**
Black Non-Hispanic	0.855	0.307	0.898	0.441	**0.697**	**0.016**	1.051	0.736	0.962	0.800
Black Hispanic	1.072	0.781	0.910	0.725	0.920	0.786	**1.678**	**0.030**	**2.047**	**0.017**
Asian/Pacific Islander	0.823	0.472	1.454	0.114	1.185	0.518	1.466	0.078	0.889	0.613
Other	0.864	0.547	1.356	0.228	1.341	0.232	0.946	0.814	1.088	0.708
Age (in 10 years)	0.973	0.552	**0.903**	**0.020**	0.925	0.088	1.010	0.814	**0.878**	**0.002**
Highest education										
High school/GED	1.000	NA	1.000	NA	1.000	NA	1.000	NA	1.000	NA
Below high school	1.098	0.710	1.139	0.587	**2.230**	**0.004**	1.483	0.091	1.213	0.466
Some college	**1.477**	**0.001**	1.229	0.054	**1.461**	**0.001**	0.924	0.447	1.135	0.242
Associates/Bachelors	**1.342**	**0.014**	1.186	0.117	1.032	0.786	0.929	0.492	0.973	0.804
Master’s degree	1.350	0.082	1.033	0.846	0.868	0.381	0.861	0.346	0.853	0.336
Doctoral degree	1.551	0.134	0.931	0.799	1.011	0.969	0.694	0.193	1.052	0.865
Marital status										
Single	1.000	NA	1.000	NA	1.000	NA	1.000	NA	1.000	NA
Married/Living with partner	1.056	0.573	1.163	0.095	1.152	0.139	**1.240**	**0.014**	1.157	0.106
Divorced/Separated/Widowed	0.927	0.591	1.154	0.292	0.982	0.897	1.198	0.180	1.172	0.250
Employment										
Employed	1.000	NA	1.000	NA	1.000	NA	1.000	NA	1.000	NA
Unemployed/Other	0.859	0.281	0.965	0.796	0.992	0.955	1.075	0.569	**0.721**	**0.013**
Disabled/Retired	0.716	0.123	0.820	0.314	0.897	0.593	0.718	0.079	0.812	0.285
Self-employed	1.032	0.872	1.084	0.632	0.749	0.123	0.876	0.453	1.011	0.951
Income										
$20,000-$39,999	1.000	NA	1.000	NA	1.000	NA	1.000	NA	1.000	NA
No income	0.993	0.972	0.945	0.744	0.932	0.696	0.930	0.676	1.064	0.719
$1-$19,999	**0.759**	**0.024**	0.853	0.157	0.970	0.801	**0.744**	**0.008**	0.844	0.145
$40,000-$59,999	0.820	0.121	0.904	0.377	1.083	0.506	0.867	0.199	0.814	0.071
$60,000-$79,999	**0.586**	**0.001**	**0.677**	**0.008**	1.046	0.768	0.833	0.195	0.765	0.059
$80,000-$99,999	0.864	0.428	**0.604**	**0.008**	1.016	0.939	**0.571**	**0.004**	0.813	0.253
$100,000+	1.009	0.963	**0.693**	**0.045**	**1.565**	**0.020**	0.913	0.594	1.194	0.325
Medical history										
Arthritis	**1.392**	**0.044**	1.081	0.603	**1.499**	**0.016**	**1.544**	**0.003**	**1.457**	**0.015**
Asthma	**1.318**	**0.031**	**1.267**	**0.039**	1.087	0.496	1.155	0.185	1.149	0.231
Chronic pain	1.367	0.071	**1.621**	**0.002**	1.172	0.372	**1.672**	**0.001**	**1.588**	**0.004**
Diabetes	1.179	0.282	0.877	0.341	**1.376**	**0.025**	1.157	0.259	1.167	0.272
High cholesterol	1.046	0.751	1.115	0.404	1.031	0.830	1.120	0.378	0.991	0.945
High blood pressure	**1.390**	**0.011**	1.165	0.187	1.024	0.844	1.138	0.258	1.154	0.198
Migraine	**1.411**	**0.035**	**1.777**	**0.000**	1.142	0.387	1.305	0.050	**1.403**	**0.019**
Sleep disorder	0.977	0.882	**1.718**	**0.000**	0.855	0.322	1.127	0.395	1.007	0.959
Psychiatric history										
Anxiety	**1.416**	**0.005**	**1.394**	**0.002**	1.063	0.597	1.171	0.127	0.979	0.850
Depression	**1.937**	**0.000**	**2.028**	**0.000**	1.371	0.067	**1.647**	**0.001**	**1.480**	**0.011**
PTSD	**1.541**	**0.038**	**1.781**	**0.001**	1.134	0.495	1.174	0.323	1.238	0.244

Bolded values indicate p-value < 0.05.

Both linear and quadratic effects of time were significant for all categories, implying that time’s effects on the probabilities of having the symptoms were nonlinear.

Males were less likely to have all COVID-19 symptoms (except musculoskeletal symptoms) than females. White Hispanics were more likely to have neuro-psychological and miscellaneous symptoms than white non-Hispanics. Black non-Hispanics were less likely to have naso-oropharyngeal symptoms, but black Hispanics were more likely to have musculoskeletal and miscellaneous symptoms than White non-Hispanics. Baseline age was negatively associated with neuro-psychological and miscellaneous symptoms.

Those educated below high school and with some college had higher odds of naso-oropharyngeal symptoms than high school graduates. Those educated more than high school (some college and associates/bachelors) were more likely to have cardiopulmonary symptoms than high school graduates. Those married or living with a partner were more likely to have musculoskeletal symptoms than singles. Those unemployed were less likely to have miscellaneous symptoms. Those with an annual income of at least $60,000 were less likely to have neuro-psychological symptoms than those with an annual income between $20,000 and $39,999.

Among the medical conditions, arthritis was associated with higher odds of all symptoms except neuro-psychological symptoms. Chronic pain was associated with higher odds of neuro-psychological, musculoskeletal, and miscellaneous symptoms. Asthma and migraine were associated with higher odds of cardiopulmonary and neuro-psychological symptoms. Each of the following medical conditions—diabetes, high blood pressure, and sleep disorder—were associated with a single category: naso-oropharyngeal, cardiopulmonary, and neuro-psychological symptoms, respectively.

Among the psychiatric conditions, depression was associated with higher odds of all COVID-19 symptoms except naso-oropharyngeal symptoms. Both anxiety and PTSD were associated with higher odds of cardiopulmonary and neuro-psychological symptoms.

## Discussion

In this study, we aimed to investigate the types of COVID-19 symptoms, profile the symptoms over time, and assess potential factors relevant to the symptoms. Our association study is different from past studies of Long COVID in several respects. In our GEE analyses, all individual events of symptoms contribute to model estimation, and this could increase the power to detect any associations. In contrast, other long COVID analyses concern only the events that meet a specific definition of long COVID. In addition, we could differentiate the effects of the risk factors according to different types of symptoms. Our GEE also focused on the population burden of specific symptoms over time, while other long COVID studies have tracked each individual in terms of how long they continue to experience any symptoms for a limited period of time.

Our study demonstrated that time, female gender, race/ethnicity, and physical and psychiatric history were associated with most of the symptoms. However, the associations were heterogeneous by the types of symptoms. For example, naso-oropharyngeal symptoms were the most affected by time with ORs of 0.229 and 1.208 for the linear and quadratic time effects. Meanwhile, neuro-psychological symptoms were the least affected with the corresponding ORs of 0.787 and 1.025. In addition, naso-oropharyngeal symptoms were associated with demographic characteristics and medical history but not with any psychiatric history. This was in contrast to other symptoms that had associations with depression. Particularly, cardiopulmonary and neuro-psychological symptoms had associations with all psychiatric conditions we considered. Another heterogeneous finding was observed for income: there was a monotonic association between annual income and neuro-psychological symptoms, with a yearly income over $60,000 being associated with the decreased odds. However, there were non-monotonic relationships between yearly income and cardiopulmonary and musculoskeletal symptoms: a lower income less than $20,000 and an income between $60,000 and $100,000 were associated with the decreased odds when compared to an income between $20,000 and $40,000. Marital status was only the significant factor for musculoskeletal symptoms; being married or living with a partner was associated with the increased odds.

In our study, female gender was associated with increased odds of cardiopulmonary, neuro-psychological, naso-oropharyngeal, and miscellaneous symptoms. Many studies demonstrated that females were more susceptible to complications after COVID-19 infection. For example, Mazza et al. [14] and Calabria et al. [15] demonstrated that female gender is associated with physical fatigue over time. Additionally, Perlis et al. [24] and Durstenfeld et al. [17] showed that female gender is associated with the development of long COVID. Lau et al. [25] found that female gender is associated with higher disability due to long COVID. A review study by Vanderlind et al. [16] and a meta-analysis study by Wang et al. [13] also demonstrated that female gender is an emerging risk factor for psychiatric symptoms among COVID-19 survivors. Consistent with these previous findings, the effect size of female gender in our study was the greatest for cardiopulmonary and neuro-psychological symptoms.

Our study showed that Hispanics were more likely to suffer COVID symptoms than white non-Hispanics: white Hispanics were more likely to have neuro-psychological and miscellaneous symptoms, and black Hispanics were more likely to experience musculoskeletal and miscellaneous symptoms. In addition, lower income was strongly associated with higher odds of neuro-psychological symptoms in our study, consistent with the meta-analysis [13] showing that lower income is associated with higher anxiety odds. The same meta-analysis showed that current employment is associated with lower odds of psychological distress, while employment was not a significant factor for neuro-psychological symptoms in our study. This might be because the pre-existing psychiatric conditions were strongly associated with neuro-psychological symptoms, and these factors could account for the effects of employment in our GEE analysis. Even though some studies [17, 26] did not find significant associations for education, our study found that education was associated with cardiopulmonary and naso-oropharyngeal symptoms. Our results for education were consistent with Perlis et al. [24], showing that some college education was associated with higher odds of the symptoms than high school education.

Many studies examined pre-existing physical conditions or medication history as risk factors for COVID symptoms [7, 13, 17, 27]. The contribution of our study is to distinguish the associations of diverse medical conditions by the types of symptoms. For example, the following non-overlapped medical conditions contributed to neuro-psychological and naso-oropharyngeal symptoms: asthma, chronic pain, migraine, and sleep disorder were significant factors for neuro-psychological, while arthritis and diabetes were the risk factors for naso-oropharyngeal symptoms. Durstenfeld et al. [17] correlated several medical conditions preceding COVID infection with long COVID. In their study, asthma and sleep disorder were not significant; however, in our study, these factors were significantly associated with cardiopulmonary and neuro-psychological symptoms.

We examined psychiatric history, including anxiety, depression, and PTSD, as risk factors of COVID-19 symptoms. Among these, depression was the strongest predictor of COVID-19 symptoms, which was significantly associated with higher odds of cardiopulmonary, neuro-psychological, musculoskeletal, and miscellaneous symptoms. This finding is consistent with those from the previous studies (Calabria et al. [15], Krishnan et al. [28], Mazza et al. [14], Townsend et al. [29], and Vanderlind et al. [16]). Durstenfeld et al. [17] adjusted the analysis for medical and psychiatric history and showed that pre-existing depression was associated with prevalent long COVID symptoms with an OR of 1.08. In our study, however, depression had the greater effect sizes (OR range: 1.41 to 1.80).

Prior studies demonstrated that the factors associated with long COVID symptoms include age, gender, race/ethnicity, income, education, urbanicity, comorbidity, psychiatric history, disease severity, vaccination, and SARS-CoV-2 variants. We included all available variables among these factors from our data in the regression models to account for any confounding as much as possible. Specifically, we considered seven sociodemographic factors and eleven pre-existing medical and psychiatric conditions. However, we were not able to account for the effects of disease severity, vaccination, and SARS-CoV-2 variants due to a lack of data. Therefore, our regression estimates could be systematically changed if these three factors are additionally adjusted for if they are significantly correlated with our model covariates.

There are several other limitations worth noting in this study. First, each model outcome represented whether at least one symptom within the category was observed but this could lead to overestimation of some of the category symptoms. Second, some participants were lost to follow-up, which could introduce bias to the regression estimates. We tracked the demographic data from baseline over 6 months and found that the respondents at follow-up were more likely to be males, White non-Hispanic, divorced/separated/widowed, and to have higher education than those at baseline (S1 Table). Third, recent studies revealed that the emergence of new variants of the SARS-CoV-2 have not only posed significant challenges in diagnostics, treatment, and vaccine efficacy but also have been associated with different phenotypes and levels of risk of developing COVID-19 symptoms [27, 30–34]. Particularly, the Omicron variant was associated with a reduced risk of long COVID development and fewer symptoms [24, 30, 35]. Our study did not measure the variants with which the participants were infected, and therefore was not able to study the associations of the variants with COVID-19 symptoms. Fourth, our sample was from one city, thus the results could not be generalized to other populations with significantly different characteristics from ours. Fifth, we did not assess the effect of vaccination on COVID-19 symptoms due to a lack of reliable vaccination data. However, the strengths of this study counterbalance these limitations: a longitudinal examination of COVID symptoms, the inclusion of various sociodemographic, pre-existing medical and psychiatric conditions, and use of an ethnically diverse population.

## Conclusion

COVID-19 symptoms are heterogeneous in that the symptoms are expressed in different body organs. Therefore, our approach using the categorical system may be useful to consider for COVID-19 symptomatology. Particularly, we demonstrated that COVID-19 symptoms were experienced differently by sociodemographic and pre-existing physical and mental conditions. Therefore, profiling high-risk individuals who develop different COVID symptoms might need attention to symptom-specific combinations or levels of risk factors. Although causal inferences cannot be made from our data, our findings suggest further investigations into the role of sex, race/ethnicity, socioeconomic status, and physical and mental health in development of Long COVID. The evaluation of the more comprehensive symptom-specific models that take into account occupation, illness severity, vaccination status, SARS-CoV-2 variants additional to the factors considered by our study merits future investigation and study.

## Supporting information

S1 Table Demographic characteristics among adults with COVID-19 infection who participated in the study at baseline, 1, 3, and 6 months. (DOCX)

S1 Checklist Human participants research checklist. (DOCX)
