# Supplementary material for: Heterogeneity of COVID-19 symptoms and associated factors: Longitudinal analysis of laboratory-confirmed COVID-19 cases in San Antonio
Source: PLoS One. 2023 Dec 8;18(12):e0295418. doi: 10.1371/journal.pone.0295418 (PMC10707584; doi:10.1371/journal.pone.0295418)
Supplement: S1 Table — (DOCX) [file pone.0295418.s001.docx]

S1 Table. Demographic characteristics among adults with COVID-19 infection who participated in the study at baseline, 1, 3, and 6 months

|  | Baseline | 1 month | 3 months | 6 months |
| --- | --- | --- | --- | --- |
|  | (N=2482) | (N=890) | (N=557) | (N=386) |
| Gender |  |  |  |  |
| Female | 1657 (66.8%) | 596 (67.0%) | 361 (64.8%) | 241 (62.4%) |
| Male | 825 (33.2%) | 294 (33.0%) | 196 (35.2%) | 145 (37.6%) |
| Race |  |  |  |  |
| White Non-Hispanic | 660 (26.6%) | 280 (31.5%) | 193 (34.7%) | 131 (33.9%) |
| White Hispanic | 1399 (56.4%) | 456 (51.2%) | 264 (47.4%) | 181 (46.9%) |
| Black Non-Hispanic | 215 (8.7%) | 79 (8.9%) | 48 (8.6%) | 35 (9.1%) |
| Black Hispanic | 58 (2.3%) | 27 (3.0%) | 20 (3.6%) | 17 (4.4%) |
| Asian/Pacific Islander | 67 (2.7%) | 26 (2.9%) | 19 (3.4%) | 13 (3.4%) |
| Other | 83 (3.3%) | 22 (2.5%) | 13 (2.3%) | 9 (2.3%) |
| Age |  |  |  |  |
| Mean (SD) | 36.1 (12.5) | 36.2 (12.5) | 37.0 (13.1) | 36.6 (13.1) |
| Median [Min, Max] | 33.0 [18.0, 84.0] | 33.0 [18.0, 84.0] | 34.0 [18.0, 84.0] | 33.0 [18.0, 72.0] |
| Education |  |  |  |  |
| High school/GED | 578 (23.3%) | 160 (18.0%) | 102 (18.3%) | 65 (16.8%) |
| Below high school | 64 (2.6%) | 20 (2.2%) | 10 (1.8%) | 10 (2.6%) |
| Some college | 762 (30.7%) | 274 (30.8%) | 162 (29.1%) | 110 (28.5%) |
| Associates/Bachelors | 804 (32.4%) | 313 (35.2%) | 204 (36.6%) | 144 (37.3%) |
| Master's degree | 223 (9.0%) | 98 (11.0%) | 70 (12.6%) | 49 (12.7%) |
| Doctoral degree | 51 (2.1%) | 25 (2.8%) | 9 (1.6%) | 8 (2.1%) |
| Marital status |  |  |  |  |
| Single | 1106 (44.6%) | 391 (43.9%) | 236 (42.4%) | 164 (42.5%) |
| Married/Living with partner | 1102 (44.4%) | 393 (44.2%) | 246 (44.2%) | 163 (42.2%) |
| D/S/W | 274 (11.0%) | 106 (11.9%) | 75 (13.5%) | 59 (15.3%) |
| Employment |  |  |  |  |
| Employed full/half-time | 1811 (73.0%) | 653 (73.4%) | 405 (72.7%) | 271 (70.2%) |
| Unemployed/Other | 400 (16.1%) | 145 (16.3%) | 84 (15.1%) | 64 (16.6%) |
| Disabled/Retired | 138 (5.6%) | 50 (5.6%) | 38 (6.8%) | 29 (7.5%) |
| Self-employed | 133 (5.4%) | 42 (4.7%) | 30 (5.4%) | 22 (5.7%) |
| Income |  |  |  |  |
| No income | 235 (9.5%) | 83 (9.3%) | 48 (8.6%) | 33 (8.5%) |
| $1-$19,999 | 535 (21.6%) | 173 (19.4%) | 103 (18.5%) | 81 (21.0%) |
| $20,000-$39,999 | 670 (27.0%) | 249 (28.0%) | 169 (30.3%) | 103 (26.7%) |
| $40,000-$59,999 | 492 (19.8%) | 180 (20.2%) | 106 (19.0%) | 77 (19.9%) |
| $60,000-$79,999 | 260 (10.5%) | 96 (10.8%) | 62 (11.1%) | 44 (11.4%) |
| $80,000-$99,999 | 128 (5.2%) | 46 (5.2%) | 32 (5.7%) | 20 (5.2%) |
| $100,000+ | 162 (6.5%) | 63 (7.1%) | 37 (6.6%) | 28 (7.3%) |

Note: D/S/W= Divorced/Separated/Widowed
